# Supplementary material for: An open-source framework for large-scale, flexible evaluation of biomedical text mining systems
Source: J Biomed Discov Collab. 2008 Jan 29;3:1. doi: 10.1186/1747-5333-3-1 (PMC2276192; doi:10.1186/1747-5333-3-1)
Supplement: Additional file 1 — Evaluation platform source code. The additional file contains the software evaluation framework discussed in this paper. The most current release can be downloaded from SourceForge.net [1]. See the accompanying README file for instructions on installation and use. Also included in the distribution is a collection of UIMA wrappers for some commonly used BioNLP tools and annotated corpora. [file 1747-5333-3-1-S1.zip › bionlp_uima_repository-1.0.1/docs/api/src-html/edu/uchsc/ccp/util/external/stanfordparser/StanfordParser_Util.html]

```
001    /*
002     *
003     * The Original Code is UCHSC Utilities.
004     *
005     * The Initial Developer of the Original Code is University of Colorado.  
006     * Copyright (C) 2005.  All Rights Reserved.
007     *
008     * UCHSC Utilities was developed by the Center for Computational Pharmacology
009     * (http://compbio.uchsc.edu) at the University of Colorado Health 
010     * Sciences Center School of Medicine with support from the National 
011     * Library of Medicine.  
012     *
013     *
014     * Contributor(s):
015     *   William A. Baumgartner <william.baumgartner@uchsc.edu> (Original Author)
016     *   
017     * Created: November 2005
018     * Dependencies:
019     *                      edu.uchsc.ccp.util.nlp.annotation.TextAnnotation;
020     *                      stanford-parser.jar
021     * ToDo: Rewrite decode using recursion.
022     * Changes:
023     *      01/10/06 - wab - Adjusting code to comply with new Type System and updated TextAnnotation class
024     *      02/19/06 - wab - Added token, phrase, and clause numbering and levels
025     *      02/22/06 - wab - Added regex escapes for the search string using GenericTextUtils
026     *                     - Rewrote to access parse tree directly instead of parsing the output format which had ambiguous parentheses
027     *                     
028     * 
029     */
030    
031    package edu.uchsc.ccp.util.external.stanfordparser;
032    
033    import java.io.StringReader;
034    import java.util.ArrayList;
035    import java.util.HashMap;
036    import java.util.Iterator;
037    import java.util.List;
038    import java.util.Set;
039    import java.util.Stack;
040    import java.util.regex.Matcher;
041    import java.util.regex.Pattern;
042    
043    import edu.stanford.nlp.ling.HasWord;
044    import edu.stanford.nlp.objectbank.TokenizerFactory;
045    import edu.stanford.nlp.parser.lexparser.LexicalizedParser;
046    import edu.stanford.nlp.parser.lexparser.Options;
047    import edu.stanford.nlp.process.DocumentPreprocessor;
048    import edu.stanford.nlp.process.Function;
049    import edu.stanford.nlp.process.WhitespaceTokenizer;
050    import edu.stanford.nlp.trees.GrammaticalStructure;
051    import edu.stanford.nlp.trees.GrammaticalStructureFactory;
052    import edu.stanford.nlp.trees.PennTreebankLanguagePack;
053    import edu.stanford.nlp.trees.Tree;
054    import edu.stanford.nlp.trees.TreeGraphNode;
055    import edu.stanford.nlp.trees.TreePrint;
056    import edu.stanford.nlp.trees.TreebankLanguagePack;
057    import edu.stanford.nlp.trees.TypedDependency;
058    import edu.stanford.nlp.trees.TypedDependencyList;
059    import edu.uchsc.ccp.util.nlp.GenericTextUtils;
060    import edu.uchsc.ccp.util.nlp.annotation.Annotator;
061    import edu.uchsc.ccp.util.nlp.annotation.TextAnnotation;
062    import edu.uchsc.ccp.util.nlp.mention.ClassMention;
063    import edu.uchsc.ccp.util.nlp.mention.ClassMentionTypes;
064    import edu.uchsc.ccp.util.nlp.mention.SlotMention;
065    import edu.uchsc.ccp.util.nlp.mention.SlotMentionTypes;
066    
067    /**
068     * 
069     * This class converts the output of the StanfordParser into a distinct text annotation objects. The main purpose for this conversion is to
070     * incorporate the StanfordParser into the UIMA framework. <br>
071     * <br>
072     * Obtain the StanfordParser here: http://nlp.stanford.edu/downloads/lex-parser.shtml
073     * 
074     * The output of this class is an ArrayList of TextAnnotations. Each TextAnnotation will have a corresponding ClassMention associated with
075     * it. The ClassMention will either be a token mention with partOfSpeech slot, a phrase with phraseType slot, or a clause mention with
076     * clauseType slot.
077     * 
078     * NOTE: Phrase/Clause Levels are incorrect.
079     * 
080     * @author William A. Baumgartner, Jr.
081     * 
082     */
083    
084    public class StanfordParser_Util {
085        private final boolean DEBUG = false;
086    
087        private LexicalizedParser lexParser; // the Stanford Parser
088    
089        private boolean showOutput = false;
090    
091        private String outputFormats = "";
092    
093        private HashMap<String, String> tagLabelsHash;
094    
095        // used to normalize the levels; 0 = base level; 4 = encomposses at least
096        // one level 3 (among others)
097        private int maxPhraseLevel = 0;
098    
099        private int maxClauseLevel = 0;
100    
101        private int tokenNumber = 0;
102    
103        private int phraseCount = 0;
104    
105        private int clauseCount = 0;
106    
107        private boolean attachTypedDependencies = false;
108        
109        private Annotator stanfordParserAnnotator;
110    
111        /* Used to keep track of where we are in the original text when we are mapping back to get the spans */
112        private int index = 0;
113    
114        /*
115         * Hash storing TokenAnnotations and their tokenNumber as key. This is used to quickly add the typed dependencies before returning the
116         * annotations.
117         */
118        private HashMap<Integer, TextAnnotation> tokenAnnotations;
119    
120        /**
121         * Construct a new StanfordParserUtil.
122         * 
123         * @param serializedGrammarFile -
124         *            Input file location. To use the PCFG parser, this file should be englishPCFG.ser.gz. To use the lexicalized parser, this
125         *            file should be englishFactored.ser.gz
126         */
127        public StanfordParser_Util(String serializedGrammarFile) {
128            initialize(serializedGrammarFile);
129        }
130    
131        public StanfordParser_Util(String serializedGrammarFile, boolean attachTypedDependencies) {
132            this.attachTypedDependencies = attachTypedDependencies;
133            initialize(serializedGrammarFile);
134        }
135        
136        /**
137         * Construct a new StanfordParserUtil while selecting the output tree display format.
138         * 
139         * @param serializedGrammarFile
140         *            Input file location. To use the PCFG parser, this file should be englishPCFG.ser.gz. To use the lexicalized parser, this
141         *            file should be englishFactored.ser.gz
142         * @param outputFormats
143         *            a comma-delimited list of StanfordParser output formats options:
144         *            penn,wordsAndTags,typedDependenciesCollapsed,oneline,words,dependencies,typedDependencies,latexTree,collocations
145         */
146        public StanfordParser_Util(String serializedGrammarFile, String outputFormats) {
147            this.outputFormats = outputFormats;
148            this.showOutput = true;
149            initialize(serializedGrammarFile);
150        }
151    
152        public StanfordParser_Util(String serializedGrammarFile, String outputFormats, boolean attachTypedDependencies) {
153            this.outputFormats = outputFormats;
154            this.showOutput = true;
155            this.attachTypedDependencies = attachTypedDependencies;
156            initialize(serializedGrammarFile);
157            
158        }
159        
160        /**
161         * Initialize the StanfordParser with the supplied input grammar file. This can take time (seconds), lexicalized parser takes longer.
162         * 
163         * @param serFile
164         */
165        private void initialize(String serializedGrammarFile) {
166            Options op = new Options();
167            lexParser = new LexicalizedParser(serializedGrammarFile, op);
168    
169            /* Initialize the default annotator */
170            stanfordParserAnnotator = new Annotator(8, "StanfordParser", "StanfordParser", "Stanford");
171    
172            // this allows the PennTreebank labels to be associated with tokens
173            // (POS), phrases, or clauses.
174            tagLabelsHash = GenericTextUtils.getPennTreebankTagLabelLookup();
175        }
176    
177        /**
178         * Call this method to parse an inputted string of text.
179         * 
180         * @param sentence
181         *            text to parse
182         */
183        public List<TextAnnotation> parse(String sentence) {
184            /* reset the max phrase level */
185            maxPhraseLevel = 0;
186    
187            // parse the input sentence
188            lexParser.parse(sentence);
189            Tree t = lexParser.getBestParse();
190    
191            if (DEBUG) {
192                System.out.println("OUTPUTTED TREE: " + t.toString());
193            }
194    
195            if (showOutput) {
196                TreePrint tp = new TreePrint(outputFormats);
197                tp.printTree(lexParser.getBestParse());
198            }
199    
200            // initialize the tokenAnnotation Hash and reinitialize the counts
201            tokenAnnotations = new HashMap<Integer, TextAnnotation>();
202            tokenNumber = 0;
203            phraseCount = 0;
204            clauseCount = 0;
205            // parse the output tree into distinct annotations
206            // return decodeParse(sentence, t.toString());
207            List<TextAnnotation> annotations = extractAnnotationsFromParseTree(sentence, t);
208    
209            if (attachTypedDependencies) {
210                assignTypedDependencies(t);
211            }
212    
213            if (DEBUG) {
214                for (TextAnnotation ta : annotations) {
215                    ta.printAnnotation(System.out);
216                }
217            }
218    
219            return annotations;
220        }
221    
222        
223    /**
224     * Not currently functional
225     * @param partiallyTaggedText
226     * @param untaggedSentence
227     * @return
228     */
229        public List<TextAnnotation> parsePartiallyTaggedText(String partiallyTaggedText, String untaggedSentence) {
230            /* reset the max phrase level */
231            maxPhraseLevel = 0;
232    
233            Options op = lexParser.getOp();
234            DocumentPreprocessor documentPreprocessor = new DocumentPreprocessor();
235            
236            
237            TokenizerFactory tokenizerFactory = WhitespaceTokenizer.factory();
238            TreebankLanguagePack tlp = op.tlpParams.treebankLanguagePack();
239    
240            documentPreprocessor.setTokenizerFactory(tokenizerFactory);
241            documentPreprocessor.setSentenceFinalPuncWords(tlp.sentenceFinalPunctuationWords());
242            documentPreprocessor.setEncoding(op.tlpParams.getInputEncoding());
243            
244            Function<List<HasWord>, List<HasWord>> escaper = null;
245            String sentenceDelimiter = null;
246            int tagDelimiter= "/".charAt(0);
247            List document = documentPreprocessor.getSentencesFromText(new StringReader(partiallyTaggedText), escaper, sentenceDelimiter, tagDelimiter);
248            
249            int count = 0;
250            for (Iterator it = document.iterator(); it.hasNext();) {
251                count++;
252                List sentence = (List) it.next();
253                lexParser.parse(sentence);
254            }
255            
256            if (count > 1) {
257                System.err.println("ERROR -- StanfordParserUtil: Stanford Parser detected multiple sentences in the input.");
258            }
259            
260            // parse the input sentence
261            Tree t = lexParser.getBestParse();
262    
263            if (DEBUG) {
264                System.out.println("OUTPUTTED TREE: " + t.toString());
265            }
266    
267            if (showOutput) {
268                TreePrint tp = new TreePrint(outputFormats);
269                tp.printTree(lexParser.getBestParse());
270            }
271    
272            // initialize the tokenAnnotation Hash and reinitialize the counts
273            tokenAnnotations = new HashMap<Integer, TextAnnotation>();
274            tokenNumber = 0;
275            phraseCount = 0;
276            clauseCount = 0;
277            // parse the output tree into distinct annotations
278            // return decodeParse(sentence, t.toString());
279            List<TextAnnotation> annotations = extractAnnotationsFromParseTree(untaggedSentence, t);
280    
281            assignTypedDependencies(t);
282    
283            if (DEBUG) {
284                for (TextAnnotation ta : annotations) {
285                    ta.printAnnotation(System.out);
286                }
287            }
288    
289            return annotations;
290        }
291    
292        
293        /**
294         * I am not sure the best way to represent these within the frame structure. Since each typed dependency is associated with two
295         * annotations, they are not really class mentions One way to approach this is to treat them as non-complex slots and to have a String
296         * value something like: "gov:0 dep:3 rel:amod"
297         * 
298         * The reason this is an issue is that in UIMA_Util,when putting a list of TextAnnotations into a JCas, it works in a recursive fashion,
299         * so if an annotation is seen as part of a ClassMention, then it is created there. What is happening is that because we currently store
300         * the TypedDependencies as Class Mentions, some of the token mentions are being automatically created as TypedDependency mentions,
301         * which is incorrect.
302         * 
303         * For now, I will use the string representation shown above.
304         * 
305         * @param t
306         */
307        private void assignTypedDependencies(Tree t) {
308            // get the typed dependencies so they can be added to the TokenAnnotations
309            List<TypedDependencyCls> typedDependencies = extractTypedDependencies(t);
310    
311            for (TypedDependencyCls utd : typedDependencies) {
312                /* Create a new typed dependency, represented by a String taking the form: "gov:# dep:# rel:relation" */
313    
314                String tdStr = "gov:" + utd.getGovernerTokenNum() + " dep:" + utd.getDependentTokenNum() + " rel:" + utd.getRelation();
315    
316                // // create a new typedDependency class mention
317                // ClassMention cm = new ClassMention();
318                // cm.setMentionName(ClassMentionTypes.TYPED_DEPENDENCY);
319                //
320                // // create a slot for the governor token
321                // SlotMention gov_sm = new SlotMention();
322                // gov_sm.setMentionName(SlotMentionTypes.TYPEDEPENDENCY_GOVERNOR_TOKEN);
323                // gov_sm.addSlotValue(new Integer(utd.getGovernerTokenNum()));
324                //
325                // // create a slot for the dependent token
326                // SlotMention dep_sm = new SlotMention();
327                // dep_sm.setMentionName(SlotMentionTypes.TYPEDEPENDENCY_DEPENDENT_TOKEN);
328                // dep_sm.addSlotValue(new Integer(utd.getDependentTokenNum()));
329                //
330                // // create a slot for the relation token
331                // SlotMention rel_sm = new SlotMention();
332                // rel_sm.setMentionName(SlotMentionTypes.TYPEDEPENDENCY_RELATION);
333                // rel_sm.addSlotValue(utd.getRelation());
334                //
335                // // add the slots to the class mention
336                // cm.addSlotMention(gov_sm);
337                // cm.addSlotMention(dep_sm);
338                // cm.addSlotMention(rel_sm);
339    
340                TextAnnotation govToken = null;
341                if (tokenAnnotations.containsKey(new Integer(utd.getGovernerTokenNum()))) {
342                    govToken = tokenAnnotations.get(new Integer(utd.getGovernerTokenNum()));
343                } else {
344                    System.err.println("gov token not found in hash... token number = " + utd.getGovernerTokenNum() + "  hash size = "
345                            + tokenAnnotations.size());
346                    Set<Integer> keys = tokenAnnotations.keySet();
347                    for (Integer k : keys) {
348                        System.out.print(k + " ");
349                    }
350                    System.out.println();
351                }
352    
353                TextAnnotation depToken = null;
354                if (tokenAnnotations.containsKey(new Integer(utd.getDependentTokenNum()))) {
355                    depToken = tokenAnnotations.get(new Integer(utd.getDependentTokenNum()));
356                } else {
357                    System.err.println("dep token not found in hash... token number = " + utd.getDependentTokenNum() + "  hash size = "
358                            + tokenAnnotations.size());
359                }
360    
361                SlotMention sm = new SlotMention();
362                sm.setMentionName(SlotMentionTypes.TOKEN_TYPED_DEPENDENCY);
363                sm.addSlotValue(tdStr);
364    
365                depToken.getClassMention().addSlotMention(sm);
366                govToken.getClassMention().addSlotMention(sm);
367    
368            }
369            // /*
370            // * associate the TypedDependency with both the governor and dependent token annotation This is the first time multiple text
371            // * annotations have been associated with class mention.. will have to wait to see if problems arise
372            // *
373            // * NOTE: THIS TURNS OUT TO BE A BAD IDEA. TD's AREN'T REALLY CLASS MENTIONS
374            // */
375            // cm.addTextAnnotation(govToken);
376            // cm.addTextAnnotation(depToken);
377            //
378            // // add the typed dependency to the governor token
379            // ComplexSlotMention govTDCSM = govToken.getClassMention().getComplexSlotMentionByName(
380            // ComplexSlotMentionTypes.TOKEN_TYPED_DEPENDENCIES);
381            // if (govTDCSM == null) {
382            // ComplexSlotMention csm = new ComplexSlotMention();
383            // csm.setMentionName(ComplexSlotMentionTypes.TOKEN_TYPED_DEPENDENCIES);
384            // csm.addClassMention(cm);
385            // govToken.getClassMention().addComplexSlotMention(csm);
386            // } else {
387            // govTDCSM.addClassMention(cm);
388            // }
389            //
390            // // add the typed dependency to the dependent token
391            // ComplexSlotMention depTDCSM = depToken.getClassMention().getComplexSlotMentionByName(
392            // ComplexSlotMentionTypes.TOKEN_TYPED_DEPENDENCIES);
393            // if (depTDCSM == null) {
394            // ComplexSlotMention csm = new ComplexSlotMention();
395            // csm.setMentionName(ComplexSlotMentionTypes.TOKEN_TYPED_DEPENDENCIES);
396            // csm.addClassMention(cm);
397            // depToken.getClassMention().addComplexSlotMention(csm);
398            // } else {
399            // depTDCSM.addClassMention(cm);
400            // }
401            // }
402    
403        }
404    
405        /*
406         * Code scavenged from edu.stanford.nlp.trees.TreePrint
407         */
408        private List<TypedDependencyCls> extractTypedDependencies(Tree tree) {
409            List<TypedDependencyCls> returnDependencies = new ArrayList<TypedDependencyCls>();
410    
411            TreebankLanguagePack tlp = new PennTreebankLanguagePack();
412            GrammaticalStructureFactory gsf = tlp.grammaticalStructureFactory();
413            GrammaticalStructure gs = gsf.newGrammaticalStructure(tree);
414    
415            TypedDependencyList tdl = gs.typedDependencies();
416    
417            if (DEBUG) {
418                System.out.println("Extracting Typed Dependencies");
419                System.out.println("TypedDependencyList Size: " + tdl.size());
420            }
421            for (TypedDependency td : tdl) {
422    
423                TreeGraphNode dependency = td.dep();
424                TreeGraphNode governor = td.gov();
425                Object relation = td.reln();
426                if (DEBUG) {
427                    System.out.println(relation.toString() + "(" + governor.toOneLineString() + ", " + dependency.toOneLineString() + ")");
428                    
429    //                System.out.println("TD: " + td.toString());
430    //                System.out.println("___ dependency:" + dependency.toOneLineString());
431    //                System.out.println("___ governor:" + governor.toOneLineString());
432    //                System.out.println("___ relationClass:" + relation.getClass().getName() + " relationString: " + relation.toString());
433                }
434    
435                // subtract 1 to line up with token numbers
436                int governorToken = Integer.parseInt(governor.toOneLineString().substring(governor.toOneLineString().lastIndexOf("-") + 1)) - 1;
437                int dependencyToken = Integer.parseInt(dependency.toOneLineString()
438                        .substring(dependency.toOneLineString().lastIndexOf("-") + 1)) - 1;
439    
440                String relationStr = relation.toString();
441                // if (relation instanceof GrammaticalRelation) {
442                // GrammaticalRelation gr = (GrammaticalRelation) relation;
443                // relationStr = gr.toString();
444                // } else {
445                // System.err.println("Relation is not a GrammaticalRelation.. not sure what to do. RELATION_CLASS: " +
446                // relation.getClass().getName() + " RELATION: " + relation.toString());
447                // System.exit(0);
448                // }
449                TypedDependencyCls utd = new TypedDependencyCls(governorToken, dependencyToken, relationStr);
450                returnDependencies.add(utd);
451                if (DEBUG) {
452                    System.out.println("Created new typed dependency: " + utd.toString());
453                }
454            }
455            return returnDependencies;
456        }
457    
458        // /* Utility class for storing typed dependency information */
459        // private class UtilTypedDependency {
460        // private int governorTokenNum;
461        //
462        // private int dependentTokenNum;
463        //
464        // private String relation;
465        //
466        // public UtilTypedDependency(int governorToken, int dependentToken, String relation) {
467        // this.dependentTokenNum = dependentToken;
468        // this.governorTokenNum = governorToken;
469        // this.relation = relation;
470        // }
471        //
472        // public int getDependentTokenNum() {
473        // return dependentTokenNum;
474        // }
475        //
476        // public void setDependentTokenNum(int dependentTokenNum) {
477        // this.dependentTokenNum = dependentTokenNum;
478        // }
479        //
480        // public int getGovernerTokenNum() {
481        // return governorTokenNum;
482        // }
483        //
484        // public void setGovernerTokenNum(int governorTokenNum) {
485        // this.governorTokenNum = governorTokenNum;
486        // }
487        //
488        // public String getRelation() {
489        // return relation;
490        // }
491        //
492        // public void setRelation(String relation) {
493        // this.relation = relation;
494        // }
495        //
496        // public String toString() {
497        // return "GOV: " + governorTokenNum + " DEP: " + dependentTokenNum + " REL: " + relation;
498        // }
499        // }
500    
501        // private void playingWithParseOutput(Tree tree) {
502        // for (Object object : tree) {
503        // System.out.println("OBJECT IN TREE IS A: " + object.getClass().getName());
504        // if (object instanceof LabeledScoredTreeNode) {
505        // System.out.println("With label: " + ((LabeledScoredTreeNode) object).label().toString() + " AND HAS: "
506        // + ((LabeledScoredTreeNode) object).getChildrenAsList().size() + "CHILDREN");
507        //
508        // } else if (object instanceof LabeledScoredTreeLeaf) {
509        // System.out.println("With label: " + ((LabeledScoredTreeLeaf) object).label().toString());
510        // }
511        // }
512        //
513        // System.out.println("============================== PARSE TREE ====================================");
514        // //parseTree(tree, 0, false, false, false, true, new PrintWriter(System.out));
515        //
516        // // System.out.println("============================== TRAVERSE TREE
517        // // ====================================");
518        // // traverseTree(tree, false, false, false, true, new
519        // // PrintWriter(System.out));
520        //
521        // }
522        //
523        // private void parseTree(Tree t, int indent, boolean parentLabelNull, boolean firstSibling, boolean leftSiblingPreTerminal,
524        // boolean topLevel, PrintWriter pw) {
525        // // the condition for staying on the same line in Penn Treebank
526        // boolean suppressIndent = (parentLabelNull || (firstSibling && t.isPreTerminal()) || (leftSiblingPreTerminal && t.isPreTerminal() &&
527        // (t
528        // .label() == null || !t.label().toString().startsWith("CC"))));
529        // if (suppressIndent) {
530        // pw.print(" ");
531        // // pw.flush();
532        // } else {
533        // if (!topLevel) {
534        // pw.println();
535        // }
536        // for (int i = 0; i < indent; i++) {
537        // pw.print(" ");
538        // // pw.flush();
539        // }
540        // }
541        // if (t.isLeaf() || t.isPreTerminal()) {
542        // pw.print(t.toString());
543        // pw.flush();
544        // return;
545        // }
546        // pw.print("(");
547        // pw.print(t.nodeString());
548        // // pw.flush();
549        // parseChildren(t.children(), indent + 1, t.label() == null || t.label().toString() == null, pw);
550        // pw.print(")");
551        // pw.flush();
552        // }
553        //
554        // private void parseChildren(Tree[] trChildren, int indent, boolean parentLabelNull, PrintWriter pw) {
555        // boolean firstSibling = true;
556        // boolean leftSibIsPreTerm = true; // counts as true at beginning
557        // for (int i = 0; i < trChildren.length; i++) {
558        // Tree currentTree = trChildren[i];
559        // parseTree(currentTree, indent, parentLabelNull, firstSibling, leftSibIsPreTerm, false, pw);
560        // leftSibIsPreTerm = trChildren[i].isPreTerminal();
561        // // CC is a special case
562        // if (trChildren[i].label() != null && trChildren[i].label().toString().startsWith("CC")) {
563        // leftSibIsPreTerm = false;
564        // }
565        // firstSibling = false;
566        // }
567        // }
568    
569        /*
570         * Modified from edu.stanford.nlp.trees.Tree.display()
571         */
572        private void traverseTree(Tree t, List<TextAnnotation> textAnnotations, Stack<TextAnnotation> annotationStack, String originalString) {
573            if (t.isLeaf() || t.isPreTerminal()) {
574                openAnnotation(originalString, annotationStack);
575                closeTokenAnnotation(t, originalString, textAnnotations, annotationStack);
576                return;
577            }
578            openAnnotation(originalString, annotationStack);
579            traverseChildren(t.children(), textAnnotations, annotationStack, originalString);
580            closePhraseClauseAnnotation(t, originalString, textAnnotations, annotationStack);
581        }
582    
583        /*
584         * This method deals with some of the ideosyncrasies found in the StanfordParser output
585         */
586        private String createSearchRegEx(String inputStr) {
587    
588            if (inputStr.contains("\\/")) {
589                inputStr = inputStr.replace("\\/", "/");
590            } else if (inputStr.equals("''")) {
591                inputStr = "\"";
592            } else if (inputStr.equals("``")) {
593                inputStr = "\"";
594            } else if (inputStr.contains("\\*")) {
595                inputStr = inputStr.replace("\\*", "*");
596            }
597    
598            /* Round brackets seem to be used to symbolize any bracket */
599            if (inputStr.equals("-LRB-")) {
600                return "[" + GenericTextUtils.escapeCharacterForRegEx("(<[{") + "]";
601            } else if (inputStr.equals("-RRB-")) {
602                return "[" + GenericTextUtils.escapeCharacterForRegEx(")]}>") + "]";
603            }
604            // else if (inputStr.equals("-LSB-")) {
605            // return "[";
606            // } else if (inputStr.equals("-RSB-")) {
607            // return "]";
608            // } else if (inputStr.equals("-LCB-")) {
609            // return "{";
610            // } else if (inputStr.equals("-RCB-")) {
611            // return "}";
612            // }
613            else {
614                return GenericTextUtils.escapeCharacterForRegEx(inputStr);
615            }
616        }
617    
618        private String getPOSLabel(String inputStr, TextAnnotation ta) {
619            /*
620             * Return the actual bracket if it is one, otherwise, just return the POS
621             */
622            if (inputStr.equals("-LRB-")) {
623                return ta.getCoveredText();
624            } else if (inputStr.equals("-RRB-")) {
625                return ta.getCoveredText();
626            } else if (inputStr.equals("SYM")) {
627                return ta.getCoveredText();
628            } else if (inputStr.equals("''")) {
629                return "\"";
630            } else if (inputStr.equals("``")) {
631                return "\"";
632            } else {
633                return inputStr;
634            }
635    
636        }
637    
638        private void closeTokenAnnotation(Tree tree, String originalString, List<TextAnnotation> textAnnotations,
639                Stack<TextAnnotation> annotationStack) {
640            if (DEBUG) {
641                System.out.println("CLOSE TOKEN: " + tree.toString() + "  LABEL: " + tree.label().toString() + "  VALUE: " + tree.value()
642                        + "  YIELD: " + tree.yield().toString());
643            }
644            TextAnnotation ta = annotationStack.pop();
645    
646            String word = tree.yield().toString();
647    
648            // //word = handleSpecialPennSymbols(word);
649            //                
650            // /**
651            // * Make custom changes here.. \/ for instance
652            // */
653            //        
654            // if (word.contains("\\/")) {
655            // word = word.replace("\\/", "/");
656            // }
657            //        
658            // // format regex properly by escaping special characters
659            // String searchStr = GenericTextUtils.escapeCharacterForRegEx(word);
660    
661            String searchStr = createSearchRegEx(word);
662    
663            Pattern searchPattern = Pattern.compile(searchStr);
664            Matcher origSentenceMatcher = searchPattern.matcher(originalString);
665            if (origSentenceMatcher.find(index)) {
666                // update index to reflect the end position of the found
667                // word
668                index = origSentenceMatcher.end();
669            } else {
670                // if we don't find the word, something is wrong, so ERROR
671                // System.err.println("Error, original text not found in original
672                // sentence, while decoding Stanford Parse tree. Word = "
673                // + word + " SearchStr = " + searchStr + " originalSentence = " +
674                // originalString);
675    
676                if (searchStr.length() > 5) {
677                    /*
678                     * make a search string that consists of the first 4 letters and last letter of the word This is done because the
679                     * StanfordParser changes some "English" spelings. homologue = homolog leukaemia = leukemia
680                     */
681                    searchStr = searchStr.substring(0, 4) + "[\\w]*" + searchStr.substring(searchStr.length() - 1, searchStr.length());
682                }
683    
684                searchPattern = Pattern.compile(searchStr);
685                origSentenceMatcher = searchPattern.matcher(originalString);
686                if (origSentenceMatcher.find(index)) {
687                    index = origSentenceMatcher.end();
688                    System.err.println("NOTE -- StanfordParserUtil: Found refactored word: " + searchStr + " for word: " + word);
689                } else {
690    
691                    // System.err.println("Error, still cannot find original text
692                    // not found in original sentence, while decoding Stanford Parse
693                    // tree. Word = "
694                    // + word + " SearchStr = " + searchStr + " originalSentence = "
695                    // + originalString);
696    
697                    /*
698                     * make a search string that consists of the first letter and last letter of the word This is done because the
699                     * StanfordParser changes some "English" spelings. homologue = homolog leukaemia = leukemia
700                     */
701                    /*
702                     * Note, I think this is inherently dangerous because it could easly match other words in the sentence. If it reaches this
703                     * step, it really needs to be checked manually for correctness.
704                     */
705                    searchStr = searchStr.substring(0, 1) + "[\\w]*" + searchStr.substring(searchStr.length() - 1, searchStr.length());
706                    searchPattern = Pattern.compile(searchStr);
707                    origSentenceMatcher = searchPattern.matcher(originalString);
708                    if (origSentenceMatcher.find(index)) {
709                        index = origSentenceMatcher.end();
710                        System.err.println("Found refactored word: " + searchStr
711                                + "   Please check manually as the match was only forced to match the first and last letters.");
712                    } else {
713                        System.err
714                                .println("Error, again, still cannot find original text not found in original sentence, while decoding Stanford Parse tree. Word = "
715                                        + word + "  SearchStr = " + searchStr + "  originalSentence = " + originalString);
716                    }
717    
718                }
719    
720            }
721    
722            /* Make sure it's supposed to be a token */
723            boolean isToken = false;
724            if (tagLabelsHash.containsKey(tree.value())) {
725                String level = tagLabelsHash.get(tree.value());
726                if (level.equalsIgnoreCase(ClassMentionTypes.TOKEN)) {
727                    isToken = true;
728                } else {
729                    System.err.println("This should be a POS tag, and it is not. Tag = " + tree.value());
730                }
731            } else {
732                // label is not in hash.. could be error, or could be
733                // punctuation, default to making it a token
734                if (DEBUG) {
735                    System.err.println("LABEL NOT FOUND: " + tree.value()
736                            + ". By default, this will treated as a POS tag and made into a token.");
737                }
738                isToken = true;
739            }
740    
741            if (isToken) {
742                ClassMention cm = new ClassMention();
743                cm.setMentionName(ClassMentionTypes.TOKEN);
744                SlotMention pos_sm = new SlotMention();
745                pos_sm.setMentionName(SlotMentionTypes.TOKEN_PARTOFSPEECH);
746    
747                cm.addSlotMention(pos_sm);
748                SlotMention toknum_sm = new SlotMention();
749                toknum_sm.setMentionName(SlotMentionTypes.TOKEN_NUMBER);
750                toknum_sm.addSlotValue(new Integer(tokenNumber++));
751                cm.addSlotMention(toknum_sm);
752                ta.setClassMention(cm);
753                closeAnnotation(ta, originalString, textAnnotations);
754                pos_sm.addSlotValue(getPOSLabel(tree.value(), ta));
755    
756                // add it to the tokenAnnotation Hash so that it can be accessed quickly later on when assigning typed dependencies
757                tokenAnnotations.put((Integer) toknum_sm.getFirstSlotValue(), ta);
758            } else {
759                System.err.println("This should be a token, but was a : " + tree.value());
760            }
761    
762        }
763    
764        private void closePhraseClauseAnnotation(Tree tree, String originalString, List<TextAnnotation> textAnnotations,
765                Stack<TextAnnotation> annotationStack) {
766            if (DEBUG) {
767                System.out.println("CLOSE PHRASE/CLAUSE: " + tree.label().toString());
768            }
769    
770            TextAnnotation ta = annotationStack.pop();
771    
772            boolean isPhrase = false;
773            boolean isClause = false;
774            if (tagLabelsHash.containsKey(tree.value())) {
775                String level = (String) tagLabelsHash.get(tree.value());
776                if (level.equalsIgnoreCase(ClassMentionTypes.CLAUSE)) {
777                    isClause = true;
778                } else if (level.equalsIgnoreCase(ClassMentionTypes.PHRASE)) {
779                    isPhrase = true;
780                } else {
781                    System.err.println("ERROR, this is not a phrase or clause. label = " + tree.value());
782                }
783            } else {
784                System.err.println("ERROR LABEL NOT FOUND: " + tree.value());
785            }
786    
787            if (isClause) {
788                ClassMention cm = new ClassMention();
789                cm.setMentionName(ClassMentionTypes.CLAUSE);
790                SlotMention sm = new SlotMention();
791                sm.setMentionName(SlotMentionTypes.CLAUSE_TYPE);
792                sm.addSlotValue(tree.value());
793                cm.addSlotMention(sm);
794                SlotMention clauseLevel_sm = new SlotMention();
795                clauseLevel_sm.setMentionName(SlotMentionTypes.CLAUSE_LEVEL);
796                clauseLevel_sm.addSlotValue(new Integer(-1));
797                cm.addSlotMention(clauseLevel_sm);
798                SlotMention clauseNum_sm = new SlotMention();
799                clauseNum_sm.setMentionName(SlotMentionTypes.CLAUSE_NUMBER);
800                clauseNum_sm.addSlotValue(new Integer(clauseCount++));
801                cm.addSlotMention(clauseNum_sm);
802                ta.setClassMention(cm);
803            } else if (isPhrase) {
804                ClassMention cm = new ClassMention();
805                cm.setMentionName(ClassMentionTypes.PHRASE);
806                SlotMention sm = new SlotMention();
807                sm.setMentionName(SlotMentionTypes.PHRASE_TYPE);
808                sm.addSlotValue(tree.value());
809                cm.addSlotMention(sm);
810                SlotMention phraseLevel_sm = new SlotMention();
811                phraseLevel_sm.setMentionName(SlotMentionTypes.PHRASE_LEVEL);
812                phraseLevel_sm.addSlotValue(new Integer(-1));
813                cm.addSlotMention(phraseLevel_sm);
814                SlotMention phraseNum_sm = new SlotMention();
815                phraseNum_sm.setMentionName(SlotMentionTypes.PHRASE_NUMBER);
816                phraseNum_sm.addSlotValue(new Integer(phraseCount++));
817                cm.addSlotMention(phraseNum_sm);
818                ta.setClassMention(cm);
819            } else {
820                System.err.println("Did not create ClassMention for bracketLabel: " + tree.value());
821            }
822    
823            closeAnnotation(ta, originalString, textAnnotations);
824        }
825    
826        private void openAnnotation(String originalString, Stack<TextAnnotation> annotationStack) {
827            // initialize a new TextAnnotation
828            TextAnnotation ta = new TextAnnotation();
829    
830            /* Set the annotator */
831            ta.setAnnotator(stanfordParserAnnotator);
832    
833            // ensure starting index is not on a space, then set it
834            try {
835            if (originalString.charAt(index) == ' ') {
836                index++;
837            }
838            } catch (StringIndexOutOfBoundsException siobe) {
839                System.err.println("StringIndexOutOfBoundsException!!!!!!!!!!!!!!!!!!!!1");
840                System.err.println("Original string = " + originalString);
841                System.err.println("String length: " + originalString.length() + "  index: " + index);
842            }
843    
844            if (DEBUG) {
845                System.out.println("OPEN ANNOTATION: index = " + index);
846            }
847    
848                // even though we only care about the start index at this point, we
849                // must set the end index in order to avoid an InvalidSpanException.
850                // We could also just catch the exception.. I'm not sure which is
851                // best.
852                ta.setAnnotationSpanEnd(index);
853                ta.setAnnotationSpanStart(index);
854    
855            // push the newly created annotation onto the stack
856            annotationStack.push(ta);
857    
858        }
859    
860        /*
861         * This method assigns an integer level to the clauses and phrases. Level 0 indicates that the phrase/clause is a base phrase/clause,
862         * i.e. it is a minimal phrase. Level n means that a phrase/clause encompasses at least n other phrases/clauses.
863         */
864        private void assignPhraseClauseLevels(List<TextAnnotation> annotations) {
865            int maxLevel = 0;
866    
867            for (int i = 0; i < annotations.size(); i++) {
868                TextAnnotation ta = annotations.get(i);
869                if (ta.getClassMention().getMentionName().equals(ClassMentionTypes.PHRASE)
870                        | ta.getClassMention().getMentionName().equals(ClassMentionTypes.CLAUSE)) {
871                    if (DEBUG) System.out.println("TA TYPE: " + ta.getClassMention().getMentionName());
872                    int spanStart = ta.getAnnotationSpanStart();
873                    int spanEnd = ta.getAnnotationSpanEnd();
874                    /* start level at -1 because at the very least, each phrase/clause will count itself as encompassing itself */
875                    int level = -1; 
876                    for (int j = 0; j < annotations.size(); j++) {
877                        TextAnnotation compareTA = annotations.get(j);
878                        if (compareTA.getClassMention().getMentionName().equals(ClassMentionTypes.PHRASE)
879                                | compareTA.getClassMention().getMentionName().equals(ClassMentionTypes.CLAUSE)) {
880                            if (DEBUG) {
881                                System.out.println("Comparing spans: " + spanStart + " -- " + spanEnd + " to " + compareTA.getAnnotationSpanStart() + " -- " + compareTA.getAnnotationSpanEnd());
882                            }
883                            if (spanStart <= compareTA.getAnnotationSpanStart() & spanEnd >= compareTA.getAnnotationSpanEnd()) {
884                                level++;
885                                if (DEBUG) {
886                                    System.out.println("Incrementing level: " + level);
887                                }
888                            }
889                        }
890                    }
891                    if (level > maxLevel) {
892                        maxLevel++;
893                        level = maxLevel;
894                    }
895    
896                    if (ta.getClassMention().getMentionName().equals(ClassMentionTypes.PHRASE)) {
897                        ta.getClassMention().getSlotMentionByName(SlotMentionTypes.PHRASE_LEVEL).setFirstSlotValue(level);
898                    } else {
899                        ta.getClassMention().getSlotMentionByName(SlotMentionTypes.CLAUSE_LEVEL).setFirstSlotValue(level);
900                    }
901                }
902            }
903    
904        }
905    
906        private void closeAnnotation(TextAnnotation ta, String originalText, List<TextAnnotation> textAnnotations) {
907            if (DEBUG) {
908                System.out.println("CLOSE ANNOTATION: index = " + index);
909            }
910    
911                ta.setAnnotationSpanEnd(index);
912                ta.setCoveredText(originalText.substring(ta.getAnnotationSpanStart(), ta.getAnnotationSpanEnd()));
913                /*
914                 * If this is a phrase or clause mention, then we need to set the corresponding phaseLevel/clauseLevel. This is computed by
915                 * taking the difference between the clause/phrase number and the current clauseCount/phraseCount
916                 */
917    //            if (ta.getClassMention().getMentionName().equals(ClassMentionTypes.PHRASE)) {
918    //
919    //                Integer phraseNum = (Integer) ta.getClassMention().getSlotMentionByName(SlotMentionTypes.PHRASE_NUMBER).getFirstSlotValue();
920    //                Integer level = new Integer(phraseCount) - phraseNum - 1;
921    //
922    //                System.out.println("Closing phrase...................................");
923    //                System.out.println("Phrase num: " + phraseNum + " at level: " + level);
924    //
925    //                if (level > maxPhraseLevel) {
926    //                    System.out.println("Increasing maxPhraseLevel");
927    //                    maxPhraseLevel++;
928    //                    level = new Integer(maxPhraseLevel);
929    //                }
930    //                System.out.println("Converted level = " + level);
931    //                ta.getClassMention().getSlotMentionByName(SlotMentionTypes.PHRASE_LEVEL).setFirstSlotValue(level);
932    //            } else if (ta.getClassMention().getMentionName().equals(ClassMentionTypes.CLAUSE)) {
933    //                Integer clauseNum = (Integer) ta.getClassMention().getSlotMentionByName(SlotMentionTypes.CLAUSE_NUMBER).getFirstSlotValue();
934    //                Integer level = new Integer(clauseCount) - clauseNum - 1;
935    //                if (level > maxClauseLevel) {
936    //                    maxClauseLevel++;
937    //                    level = new Integer(maxClauseLevel);
938    //                }
939    //                ta.getClassMention().getSlotMentionByName(SlotMentionTypes.CLAUSE_LEVEL).setFirstSlotValue(level);
940    //            }
941    
942                textAnnotations.add(ta);
943        }
944    
945        /*
946         * Modified from edu.stanford.nlp.trees.Tree.displayChildren()
947         */
948        private void traverseChildren(Tree[] trChildren, List<TextAnnotation> textAnnotations, Stack<TextAnnotation> annotationStack,
949                String originalString) {
950            // boolean firstSibling = true;
951            // boolean leftSibIsPreTerm = true; // counts as true at beginning
952            for (int i = 0; i < trChildren.length; i++) {
953                Tree currentTree = trChildren[i];
954                traverseTree(currentTree, textAnnotations, annotationStack, originalString);
955                // leftSibIsPreTerm = trChildren[i].isPreTerminal();
956                // // CC is a special case
957                // if (trChildren[i].label() != null &&
958                // trChildren[i].label().toString().startsWith("CC")) {
959                // leftSibIsPreTerm = false;
960                // }
961                // firstSibling = false;
962            }
963        }
964    
965        private ArrayList<TextAnnotation> extractAnnotationsFromParseTree(String originalString, Tree tree) {
966            /* tokenNumber tracks token ordering in sentence */
967    
968            // stores open annotations as we go
969            Stack<TextAnnotation> stack = new Stack<TextAnnotation>();
970    
971            ArrayList<TextAnnotation> returnAnnotations = new ArrayList<TextAnnotation>();
972    
973            index = 0;
974            // int index = 0; // keeps track of where we are in the original text
975    
976            traverseTree(tree, returnAnnotations, stack, originalString);
977    
978            // for (TextAnnotation ta : returnAnnotations) {
979            // ta.printAnnotation(System.out);
980            // }
981    
982            assignPhraseClauseLevels(returnAnnotations);
983            
984            return returnAnnotations;
985    
986        }
987    
988    //    /*
989    //     * This method decodes the output of the StanfordParser, and transforms it into distinct TextAnnotations. It works from left to right,
990    //     * starting a new TextAnnotation for every "(" and closing the TextAnnotation for every ")". A stack is used to keep track of open
991    //     * TextAnnotations.
992    //     * 
993    //     * A more elegant way to write this method would be to use recursion.
994    //     */
995    //    @Deprecated
996    //    private ArrayList<TextAnnotation> decodeParse(String sentence, String treeString) {
997    //
998    //        /* tokenNumber tracks token ordering in sentence */
999    //        int tokenNumber = 0;
1000    //        int phraseCount = 0;
1001    //        int clauseCount = 0;
1002    //
1003    //        Stack<TextAnnotation> stack = new Stack<TextAnnotation>(); // stores
1004    //        // open
1005    //        // brackets
1006    //        // as we go
1007    //        ArrayList<TextAnnotation> returnAnnotations = new ArrayList<TextAnnotation>();
1008    //
1009    //        int index = 0; // keeps track of where we are in the original text
1010    //        String bracketLabel;
1011    //
1012    //        Matcher origSentenceMatcher; // used to find the index in the
1013    //        // original text
1014    //        String searchStr = "";
1015    //
1016    //        // remove all [##.##] from the tree
1017    //        treeString = treeString.replaceAll("\\[-?\\d+\\.\\d+\\]\\s", "");
1018    //        if (DEBUG) {
1019    //            System.out.println("REMOVED [##.###] FROM TREE STRING: " + treeString);
1020    //        }
1021    //
1022    //        // decode the parse tree
1023    //        while (index < sentence.length()) {
1024    //            int lbrIndex = treeString.indexOf("(");
1025    //            int rbrIndex = treeString.indexOf(")");
1026    //
1027    //            if (lbrIndex < rbrIndex) { // open a new bracket, i.e. this is the
1028    //                // start of a new clause, phrase, or token (POS)
1029    //                treeString = treeString.trim(); // make sure there are no
1030    //                // leading spaces
1031    //
1032    //                // get bracket label, i.e. "ROOT","S","NP","NNS",etc.
1033    //                bracketLabel = treeString.substring(1, treeString.indexOf(" "));
1034    //
1035    //                // get rid of bracket label plus next leading space in the parse
1036    //                // tree string
1037    //                treeString = treeString.substring(treeString.indexOf(" ") + 1);
1038    //
1039    //                if (DEBUG) {
1040    //                    System.out.println("Open bracket [" + bracketLabel + "]  StartIndex: " + index + "   TREE: " + treeString);
1041    //                }
1042    //
1043    //                // initialize a new TextAnnotation
1044    //                TextAnnotation ta = new TextAnnotation();
1045    //
1046    //                // ensure starting index is not on a space, then set it
1047    //                if (sentence.charAt(index) == ' ') {
1048    //                    index++;
1049    //                }
1050    //
1051    //                try {
1052    //                    // even though we only care about the start index at this
1053    //                    // point, we must set the end index in order to avoid an
1054    //                    // InvalidSpanException. We could also just catch the
1055    //                    // exception.. I'm not sure which is best.
1056    //                    ta.setFirstSpanEndIndex(index);
1057    //                    ta.setFirstSpanStartIndex(index);
1058    //                } catch (InvalidSpanException e) {
1059    //                    // TODO Auto-generated catch block
1060    //                    e.printStackTrace();
1061    //                }
1062    //                // ta.setBeginIndex(index);
1063    //
1064    //                // if this is a POS annotation, we need to get the text and
1065    //                // update index variable
1066    //                String firstPosition = treeString.substring(0, 1);
1067    //                String word = "";
1068    //                // check to see if this is a TokenAnnotation
1069    //                if (!(firstPosition.equals("(") | firstPosition.equals(")"))) {
1070    //                    // then this is a POS tag, and therefore represents a
1071    //                    // TokenAnnotaiton
1072    //
1073    //                    // get the word corresponding to the POS tag
1074    //                    word = treeString.substring(0, treeString.indexOf(")"));
1075    //
1076    //                    // compensate for bracket conversion used by Penn TreeBank.
1077    //                    // we must return (, ), [, ], {, and } from -LRB-, -RRB-,
1078    //                    // etc.
1079    //                    if (word.equals("-LRB-")) {
1080    //                        // remove word from treeString
1081    //                        treeString = treeString.substring(word.length());
1082    //                        word = "(";
1083    //                        bracketLabel = "(";
1084    //                        // searchStr = ("\\" + word);
1085    //                    } else if (word.equals("-RRB-")) {
1086    //                        treeString = treeString.substring(word.length());
1087    //                        word = ")";
1088    //                        bracketLabel = ")";
1089    //                        // searchStr = ("\\" + word);
1090    //                    } else if (word.equals("-LSB-")) {
1091    //                        treeString = treeString.substring(word.length());
1092    //                        word = "[";
1093    //                        bracketLabel = "[";
1094    //                        // searchStr = ("\\" + word);
1095    //                    } else if (word.equals("-RSB-")) {
1096    //                        treeString = treeString.substring(word.length());
1097    //                        word = "]";
1098    //                        bracketLabel = "]";
1099    //                        // searchStr = ("\\" + word);
1100    //                    } else if (word.equals("-LCB-")) {
1101    //                        treeString = treeString.substring(word.length());
1102    //                        word = "{";
1103    //                        bracketLabel = "{";
1104    //                        // searchStr = ("\\" + word);
1105    //                    } else if (word.equals("-RCB-")) {
1106    //                        treeString = treeString.substring(word.length());
1107    //                        word = "}";
1108    //                        bracketLabel = "}";
1109    //                        // searchStr = ("\\" + word);
1110    //                    } else {
1111    //                        treeString = treeString.substring(word.length());
1112    //                        // searchStr = word; // add the word to the search
1113    //                        // string
1114    //                        // so we can find the real end
1115    //                        // location in the original sentence
1116    //                    }
1117    //                    searchStr = word;
1118    //
1119    //                    // format regex properly by escaping special characters
1120    //                    searchStr = GenericTextUtils.escapeCharacterForRegEx(searchStr);
1121    //
1122    //                    if (DEBUG) {
1123    //                        System.out.println("WORD: " + word);
1124    //                        System.out.println("SEARCH STR: " + searchStr);
1125    //                    }
1126    //
1127    //                    // search for the word in the original text, starting from
1128    //                    // "index"
1129    //
1130    //                    Pattern searchPattern = Pattern.compile(searchStr);
1131    //                    origSentenceMatcher = searchPattern.matcher(sentence);
1132    //                    if (origSentenceMatcher.find(index)) {
1133    //                        // update index to reflect the end position of the found
1134    //                        // word
1135    //                        index = origSentenceMatcher.end();
1136    //                    } else {
1137    //                        // if we don't find the word, something is wrong, so
1138    //                        // ERROR
1139    //                        System.err
1140    //                                .println("Error, original text not found in original sentence, while decoding Stanford Parse tree. Word = "
1141    //                                        + word + "  SearchStr = " + searchStr);
1142    //                    }
1143    //                } // end -- if this is not a POS tag
1144    //
1145    //                /*
1146    //                 * The output of the Stanford Parser will a token, phrase, or clause.
1147    //                 * 
1148    //                 */
1149    //
1150    //                // ta.addClassMention(bracketLabel, word); 01/10/06 - wab -
1151    //                // Changed to comply with new Type System, and updated
1152    //                // TextAnnotation class
1153    //                boolean isToken = false;
1154    //                boolean isPhrase = false;
1155    //                boolean isClause = false;
1156    //                boolean isSentence = false;
1157    //                if (tagLabelsHash.containsKey(bracketLabel)) { // then
1158    //                    // determine if
1159    //                    // it is a
1160    //                    // clause, phrase, or token
1161    //                    String level = (String) tagLabelsHash.get(bracketLabel);
1162    //                    if (level.equalsIgnoreCase(ClassMentionTypes.CLAUSE)) {
1163    //                        isClause = true;
1164    //                    } else if (level.equalsIgnoreCase(ClassMentionTypes.PHRASE)) {
1165    //                        isPhrase = true;
1166    //                    } else if (level.equalsIgnoreCase(ClassMentionTypes.TOKEN)) {
1167    //                        isToken = true;
1168    //                    } else if (level.equalsIgnoreCase(ClassMentionTypes.SENTENCE)) {
1169    //                        isSentence = true;
1170    //                    } else {
1171    //                        if (DEBUG) {
1172    //                            System.err.println("ERROR, label not found in tagLabelsHash.");
1173    //                        }
1174    //                    }
1175    //
1176    //                } else {
1177    //                    // label is not in hash.. could be error, or could be
1178    //                    // punctuation, default to making it a token
1179    //                    if (DEBUG) {
1180    //                        System.err.println("LABEL NOT FOUND: " + bracketLabel
1181    //                                + ". By default, this will treated as a POS tag and made into a token.");
1182    //                    }
1183    //                    isToken = true;
1184    //                    // by default, create a TokenAnnotation for
1185    //                    // anything without a match
1186    //                }
1187    //
1188    //                if (isClause) {
1189    //                    ClassMention cm = new ClassMention();
1190    //                    cm.setMentionName(ClassMentionTypes.CLAUSE);
1191    //                    SlotMention sm = new SlotMention();
1192    //                    sm.setMentionName(SlotMentionTypes.CLAUSE_TYPE);
1193    //                    sm.addSlotValue(bracketLabel);
1194    //                    cm.addSlotMention(sm);
1195    //                    SlotMention clauseLevel_sm = new SlotMention();
1196    //                    clauseLevel_sm.setMentionName(SlotMentionTypes.CLAUSE_LEVEL);
1197    //                    clauseLevel_sm.addSlotValue(new Integer(-1));
1198    //                    cm.addSlotMention(clauseLevel_sm);
1199    //                    SlotMention clauseNum_sm = new SlotMention();
1200    //                    clauseNum_sm.setMentionName(SlotMentionTypes.CLAUSE_NUMBER);
1201    //                    clauseNum_sm.addSlotValue(new Integer(clauseCount++));
1202    //                    cm.addSlotMention(clauseNum_sm);
1203    //                    ta.setClassMention(cm);
1204    //                } else if (isPhrase) {
1205    //                    ClassMention cm = new ClassMention();
1206    //                    cm.setMentionName(ClassMentionTypes.PHRASE);
1207    //                    SlotMention sm = new SlotMention();
1208    //                    sm.setMentionName(SlotMentionTypes.PHRASE_TYPE);
1209    //                    sm.addSlotValue(bracketLabel);
1210    //                    cm.addSlotMention(sm);
1211    //                    SlotMention phraseLevel_sm = new SlotMention();
1212    //                    phraseLevel_sm.setMentionName(SlotMentionTypes.PHRASE_LEVEL);
1213    //                    phraseLevel_sm.addSlotValue(new Integer(-1));
1214    //                    cm.addSlotMention(phraseLevel_sm);
1215    //                    SlotMention phraseNum_sm = new SlotMention();
1216    //                    phraseNum_sm.setMentionName(SlotMentionTypes.PHRASE_NUMBER);
1217    //                    phraseNum_sm.addSlotValue(new Integer(phraseCount++));
1218    //                    cm.addSlotMention(phraseNum_sm);
1219    //                    ta.setClassMention(cm);
1220    //                } else if (isToken) {
1221    //                    ClassMention cm = new ClassMention();
1222    //                    cm.setMentionName(ClassMentionTypes.TOKEN);
1223    //                    SlotMention pos_sm = new SlotMention();
1224    //                    pos_sm.setMentionName(SlotMentionTypes.TOKEN_PARTOFSPEECH);
1225    //                    pos_sm.addSlotValue(bracketLabel);
1226    //                    cm.addSlotMention(pos_sm);
1227    //                    SlotMention toknum_sm = new SlotMention();
1228    //                    toknum_sm.setMentionName(SlotMentionTypes.TOKEN_NUMBER);
1229    //                    toknum_sm.addSlotValue(new Integer(tokenNumber++));
1230    //                    cm.addSlotMention(toknum_sm);
1231    //                    ta.setClassMention(cm);
1232    //                } else if (isSentence) {
1233    //                    ClassMention cm = new ClassMention();
1234    //                    cm.setMentionName(ClassMentionTypes.SENTENCE);
1235    //                    ta.setClassMention(cm);
1236    //                } else {
1237    //                    System.err.println("Did not create ClassMention for bracketLabel: " + bracketLabel);
1238    //                }
1239    //
1240    //                stack.push(ta);
1241    //            } else {
1242    //                // this is the end of something so, close an old bracket
1243    //                treeString = treeString.substring(1); // remove the ")"
1244    //
1245    //                TextAnnotation ta = (TextAnnotation) stack.pop();
1246    //                closeBracket(ta, index, phraseCount, clauseCount, sentence);
1247    //                // ta.setEndIndex(index);
1248    //
1249    //                // save TextAnnotation in ArrayList to be returned by method
1250    //                returnAnnotations.add(ta);
1251    //
1252    //                if (DEBUG) {
1253    //                    ClassMention cm = ta.getClassMention();
1254    //                    if (cm.getMentionName().equals(ClassMentionTypes.SENTENCE)) {
1255    //                        System.out.print("Close bracket [sentence");
1256    //                    } else {
1257    //                        System.out.print("Close bracket [" + ta.getClassMention().getFirstSlotMention().getFirstSlotValue());
1258    //                    }
1259    //                    System.out.println("]  StartIndex: " + ta.getFirstSpanStartIndex() + " EndIndex: " + ta.getFirstSpanEndIndex()
1260    //                            + "   TREE: " + treeString);
1261    //                }
1262    //
1263    //            } // end -- else close an old bracket
1264    //
1265    //        } // end -- while endIndex < sentence length
1266    //
1267    //        // pop stack until Empty to get remaining TextAnnotations
1268    //        while (!stack.isEmpty()) {
1269    //            TextAnnotation ta = (TextAnnotation) stack.pop();
1270    //            closeBracket(ta, index, phraseCount, clauseCount, sentence);
1271    //            // try {
1272    //            // ta.setFirstSpanEndIndex(index);
1273    //            // } catch (InvalidSpanException e) {
1274    //            // // TODO Auto-generated catch block
1275    //            // e.printStackTrace();
1276    //            // }
1277    //            returnAnnotations.add(ta);
1278    //            if (DEBUG) {
1279    //                ClassMention cm = ta.getClassMention();
1280    //                if (cm.getMentionName().equals(ClassMentionTypes.SENTENCE)) {
1281    //                    System.out.print("Close bracket [sentence");
1282    //                } else {
1283    //                    System.out.print("Close bracket [" + ta.getClassMention().getFirstSlotMention().getFirstSlotValue());
1284    //                }
1285    //                System.out.println("]  StartIndex: " + ta.getFirstSpanStartIndex() + " EndIndex: " + ta.getFirstSpanEndIndex()
1286    //                        + "   TREE: " + treeString);
1287    //            }
1288    //        } // end -- while !stack.isEmpty()
1289    //
1290    //        // /* Assign phrase_number, phrase_level */
1291    //        // Collections.sort(returnAnnotations);
1292    //        // int phraseLevel = 0;
1293    //        // Stack<TextAnnotation> openAnnotations = new Stack<TextAnnotation>();
1294    //        // int childrenCount = 0;
1295    //        // int spanStart = 0;
1296    //        // int spanEnd = 0;
1297    //        // for (TextAnnotation ta : returnAnnotations) {
1298    //        // if
1299    //        // (ta.getClassMention().getMentionName().equals(ClassMentionTypes.PHRASE))
1300    //        // {
1301    //        // spanStart = ta.getFirstSpanStartIndex();
1302    //        // spanEnd = ta.getFirstSpanEndIndex();
1303    //        // openAnnotations.push(ta);
1304    //        //                            
1305    //        // }
1306    //        //            
1307    //        // }
1308    //
1309    //        return returnAnnotations;
1310    //    } // end -- method decodeParse()
1311    //
1312    //    private void closeBracket(TextAnnotation ta, int index, int phraseCount, int clauseCount, String originalText) {
1313    //        try {
1314    //            ta.setFirstSpanEndIndex(index);
1315    //            ta.setCoveredText(originalText.substring(ta.getFirstSpanStartIndex(), ta.getFirstSpanEndIndex()));
1316    //            /*
1317    //             * If this is a phrase or clause mention, then we need to set the corresponding phaseLevel/clauseLevel. This is computed by
1318    //             * taking the difference between the clause/phrase number and the current clauseCount/phraseCount
1319    //             */
1320    //            if (ta.getClassMention().getMentionName().equals(ClassMentionTypes.PHRASE)) {
1321    //                Integer phraseNum = (Integer) ta.getClassMention().getSlotMentionByName(SlotMentionTypes.PHRASE_NUMBER).getFirstSlotValue();
1322    //                Integer level = new Integer(phraseCount) - phraseNum - 1;
1323    //                if (level > maxPhraseLevel) {
1324    //                    maxPhraseLevel++;
1325    //                    level = new Integer(maxPhraseLevel);
1326    //                }
1327    //                ta.getClassMention().getSlotMentionByName(SlotMentionTypes.PHRASE_LEVEL).setFirstSlotValue(level);
1328    //            } else if (ta.getClassMention().getMentionName().equals(ClassMentionTypes.CLAUSE)) {
1329    //                Integer clauseNum = (Integer) ta.getClassMention().getSlotMentionByName(SlotMentionTypes.CLAUSE_NUMBER).getFirstSlotValue();
1330    //                Integer level = new Integer(clauseCount) - clauseNum - 1;
1331    //                if (level > maxClauseLevel) {
1332    //                    maxClauseLevel++;
1333    //                    level = new Integer(maxClauseLevel);
1334    //                }
1335    //                ta.getClassMention().getSlotMentionByName(SlotMentionTypes.CLAUSE_LEVEL).setFirstSlotValue(level);
1336    //            }
1337    //        } catch (InvalidSpanException e) {
1338    //            // TODO Auto-generated catch block
1339    //            e.printStackTrace();
1340    //        }
1341    //    }
1342    
1343        /**
1344         * @param args
1345         *            args[0] = serialized grammar file <br>
1346         *            args[1] = text to parse <br>
1347         *            args[2] = [OPTIONAL] output tree display format <br>
1348         */
1349        public static void main(String[] args) {
1350            StanfordParser_Util spu;
1351            if (args.length == 2) {
1352                spu = new StanfordParser_Util(args[0]);
1353            } else {
1354                spu = new StanfordParser_Util(args[0], args[2]);
1355            }
1356            List<TextAnnotation> textAnnotations = spu.parse(args[1]);
1357            for (TextAnnotation ta : textAnnotations) {
1358                ta.printAnnotation(System.out);
1359            }
1360        }
1361    
1362    }
```
